# Combining in vivo and in vitro biomechanical data reveals key roles of perivascular tethering in central artery function

**DOI:** 10.1371/journal.pone.0201379

**Published:** 2018-09-07

**Authors:** Jacopo Ferruzzi, Paolo Di Achille, George Tellides, Jay D. Humphrey

**Affiliations:** 1 Department of Biomedical Engineering, Yale University, New Haven, CT, United States of America; 2 Department of Surgery, Yale School of Medicine, New Haven, CT, United States of America; 3 Vascular Biology and Therapeutics Program, Yale School of Medicine, New Haven, CT, United States of America; Medical University Innsbruck, AUSTRIA

## Abstract

Considerable insight into effectors of cardiovascular function can be gleaned from controlled studies on mice, especially given the diverse models that are available. Toward this end, however, there is a need for consistent and complementary methods of in vivo and in vitro data analysis, synthesis, and interpretation. The overall objective of this study is twofold. First, we present new semi-automated methods to quantify in vivo measurements of vascular function in anesthetized mice as well as new approaches to synthesize these data with those from in vitro biaxial mechanical characterizations. Second, we contrast regional differences in biomechanical behaviors along the central vasculature by combining biaxial strains measured in vivo with data on the unloaded geometry and biaxial material properties measured in vitro. Results support the hypothesis that the healthy ascending aorta stores significant elastic energy during systole, which is available to work on the heart and blood during diastole, particularly during periods of physical exertion, and further suggest that perivascular tethering allows arteries to work at lower values of wall stress and material stiffness than often assumed. The numerous measurements of vascular function and properties provided herein can also serve as reference values for normal wild-type male and female mice, to which values for myriad genetic, surgical, and pharmacological models can be compared in future studies.

## Introduction

Central arteries stiffen during normal aging as well as with diabetes, particular genetic disorders, hypertension, and multiple pathologies including aneurysm. Increased stiffness affects local arterial function (e.g., cell phenotype and matrix composition) and global hemodynamics (e.g., central blood pressure and flow), both mediated in part by complex interactions between the central and peripheral circulations [1]. Arterial stiffening typically associates with the disorganization, damage, or degradation of elastin and/or remodeling of fibrillar collagen, including increased deposition and cross-linking [2]. The former reduces arterial elasticity, which compromises the cushioning function (often described via a Windkessel model); the latter limits arterial distensibility, which compromises the conduit function (often described via wave propagation models). Both effects cause the pressure wave to propagate faster towards and deeper into the peripheral circulation [3,4]. This increased pulse wave velocity (PWV) leads to central pulse pressure augmentation and microcirculatory damage in end organs [5–7].

PWV is often measured via applanation tonometry at carotid and femoral sites, thus yielding the cf-PWV that has become the clinical gold standard measure of central artery stiffness. Yet, cf-PWV is a global metric that integrates individual contributions of heterogeneous arterial segments from the carotid to the femoral and it cannot delineate even the normal acceleration of the pressure pulse wave as it travels down the aorta [8]. Ironically, cf-PWV measurements also exclude the ascending aorta, which can experience the greatest changes in aortic geometry with aging, including a progressively enlarging diameter and increasing axial length (e.g., nearly two-fold lengthening between 20 and 80 years of age) [9].

Local measurements of stiffness in vivo have the potential to delineate regional heterogeneities in central artery properties, perhaps allowing an earlier detection of compromised vascular functionality. Preferred methods would exploit noninvasive imaging, including ultrasound or magnetic resonance imaging (MRI). In particular, Doppler ultrasound and phase contrast MRI can estimate regional PWVs assuming that pressure and flow pulses propagate at the same velocity [10]. Despite its higher cost, MRI has the added advantage of quantifying the path length traveled by the pulse wave [11], which often increases with aging and disease and manifests as tortuosity. Local distensibility *D*, or circumferential strain *ε* (where *D* = *ε*/(*P*_sys_ − *P*_dias_), with $\varepsilon =(d_{sys}^{n}-d_{dias}^{n})/{nd}_{dias}^{n}$ for *n* = 1 or 2, *P* denoting luminal pressure, and *d* luminal diameter), can be sensitive markers of structural stiffening [12,13]. The luminal diameter of the proximal aorta is assessed easily in transthoracic or transesophageal M-Mode ultrasound [14], although associated calculations of distensibility can be compromised by using brachial, not central, pulse pressure. Despite this limitation, local assessment of thoracic aortic distensibility via ultrasound is thought to be comparable to PWV in terms of predictive ability [15].

Our ultimate goal is to understand and improve human cardiovascular health, but the wide availability of genetic, pharmacologic, and surgical mouse models has rendered mice the species of choice in many cardiovascular studies. The objective of this study is twofold. First, we present new semi-automated methods to quantify in vivo measurements of vascular mechanics in anesthetized mice as well as new approaches to synthesize these data with those from in vitro mechanical testing. Second, we contrast regional differences in biomechanical behaviors along the central vasculature by combining biaxial strains measured in vivo with data on the unloaded geometry and biaxial material properties measured in vitro. One hypothesis is that, due to its high elasticity and physical connection with the beating heart, the ascending aorta plays a particularly important role in determining overall cardiovascular function. Indeed, we found that, among four key central arteries, the ascending aorta stores the most elastic energy during a cardiac cycle, which it can then use to work on both the blood and the heart. Finally, we show that including effects of perivascular tethering, which can be estimated by combining in vivo and in vitro biomechanical data, suggests that arteries operate in vivo at lower values of wall stress and thus material stiffness than often assumed. The role of perivascular tethering of central arteries in overall cardiovascular function merits increased attention.

## Methods and materials

### General approach

All animal procedures were approved by the Institutional Animal Care and Use Committee (IACUC) of Yale University. In vivo data were collected from 13 adult (20.9±0.2 weeks of age) wild-type (C57BL/6 x 129/SvEv) mice of both sexes (5 males, 8 females). Specifically, noninvasive ultrasound data were collected using a high-frequency Vevo 2100 system (Visualsonics, Toronto, Canada) with a linear array probe (MS550D, 22–55 MHz) whereas invasive pressure data were collected using a Millar SPR-1000 catheter (Millar, Houston, TX) with a 1F outer diameter. Custom data analysis software developed in Matlab R2016a enabled semi-automatic quantification of many in vivo metrics of interest, as detailed below. Finally, previously collected in vitro biomechanical data from 10 adult (21.1±0.2 weeks of age) wild-type mice of both sexes (5 males, 5 females) on the same background [16] were used to interpret the in vivo data further, as described below.

### Anesthesia

Animals were placed in an induction chamber and exposed to 2.0–3.0% isoflurane mixed with 0.6 L/min of 100% O_2_. Once anesthetized, the mice were transferred to an ultrasound platform where they received 1.5% isoflurane to maintain a steady-state level throughout the in vivo study. If animals showed signs of distress, isoflurane was increased up to 2% while keeping oxygen constant at 0.6 L/min. Compared with conscious conditions, 1.5–2.0% isoflurane causes modest reductions in heart rate and mean arterial pressure relative to other anesthetic regimens [17]. Body temperature was monitored with a rectal probe and maintained at 37° using a feedback-controlled heating platform.

### Ultrasound

**Fig 1:**
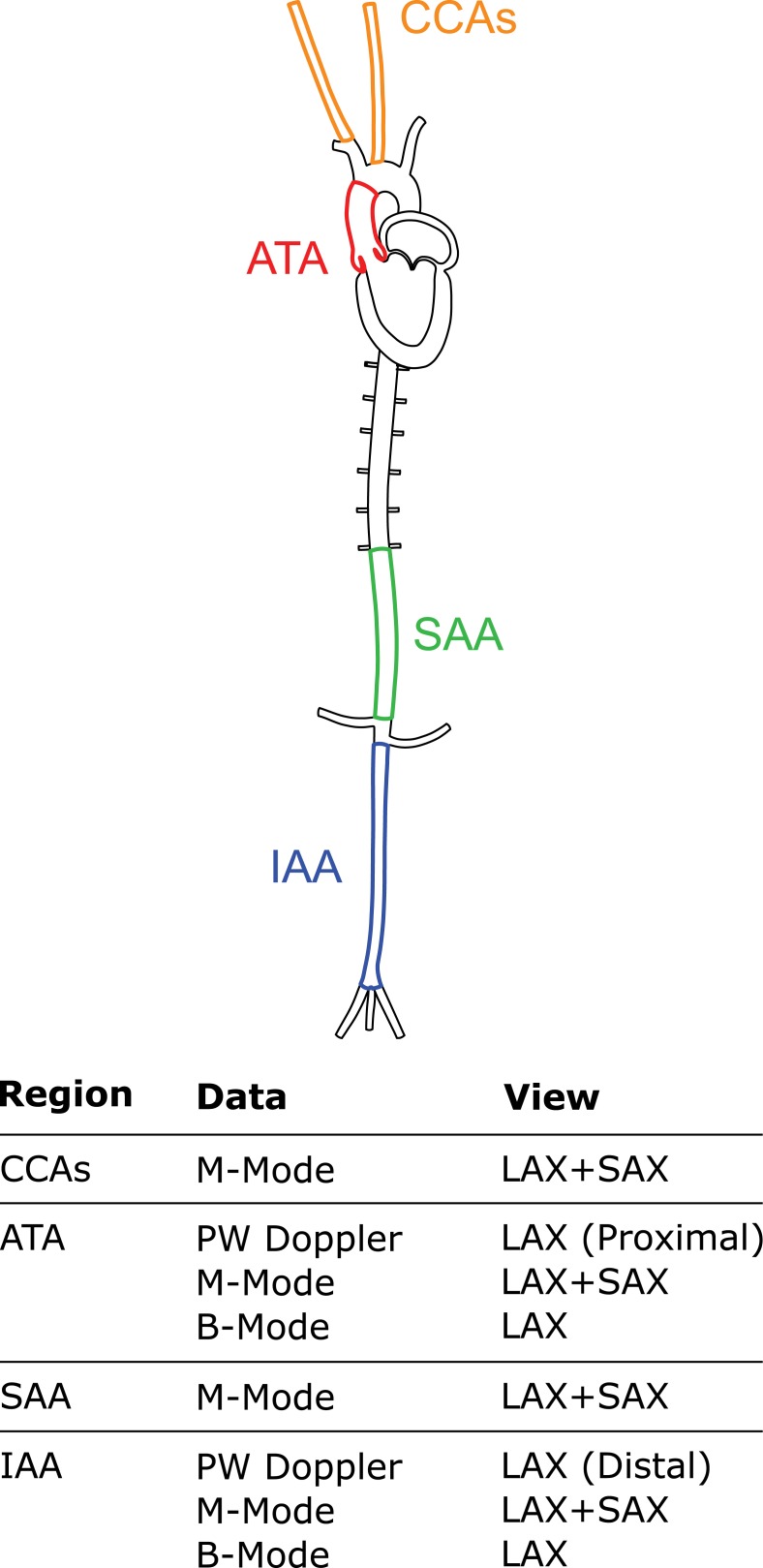
Our regional assessment of central artery function in vivo includes ultrasound measurements in the ascending thoracic aorta (ATA), suprarenal abdominal aorta (SAA), infrarenal abdominal aorta (IAA), and one of the common carotid arteries (CCAs). B-Mode imaging tracks biaxial motions in the ATA and IAA, namely inner diameter and axial length. M-Mode imaging quantifies inner diameter in all regions. PW Doppler imaging in the ATA and IAA quantifies blood velocities and pulse transit time.

Fig 1 shows the four central arterial regions of interest: the ascending thoracic aorta (ATA), suprarenal abdominal aorta (SAA), infrarenal abdominal aorta (IAA), and one common carotid artery (CCA). The proximal descending thoracic aorta, which is usually included in our in vitro biomechanical phenotyping [18], could not be imaged well due to interference from the air-filled adjacent lungs. B-Mode imaging located each segment via anatomical landmarks while M-Mode sequences measured time-varying luminal diameters in all four regions in short axis (SAX) and long axis (LAX) views. PW Doppler measured near centerline blood velocities proximally (in the ATA near the aortic root) and distally (in the IAA close to the iliac bifurcation), which enabled assessment of aortic pulse wave transit times. Finally, long axis B-Mode cine loops (80 to 340 frames/second, depending on region) captured cyclic changes in luminal diameter and axial length of the ATA and IAA. To ensure reliable axial length quantifications, we used consistent anatomic landmarks to identify each segment in each view: the ATA is delimited by the aortic root and brachiocephalic trunk while the IAA is delimited by the left renal artery and iliac bifurcation (Fig 1). We use these same anatomic landmarks to isolate vessels for in vitro biaxial testing [16], which allowed direct comparisons of in vivo and in vitro data.

### Invasive blood pressures

Preemptive analgesia was given via an intraperitoneal injection of 0.3 mg/kg meloxicam (Santa Cruz Biotechnology, Santa Cruz, CA). While under anesthesia, the right common carotid artery was punctured using a 21G needle, then a Millar SPR-1000 catheter was introduced, secured using one or two 6–0 silk sutures, and advanced to the mid-section of the ATA. B-Mode imaging enabled correct positioning and, after verifying stable waveforms, continuous pressure data were recorded over multiple cycles.

### In vivo data analysis

**Fig 2:**
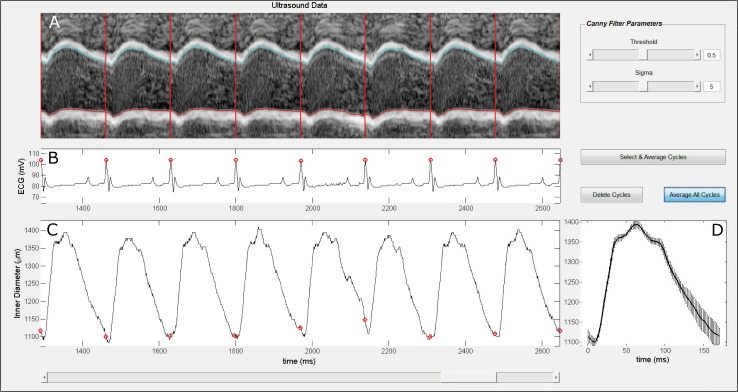
Graphical user interface (GUI) for semi-automated M-Mode data analysis. The GUI visualizes eight cardiac cycles at a time (A) while allowing the user to examine the entire series of data via a slider (bottom). Canny filter parameters (top-right) can be tuned to detect the inner diameter of the vessel from M-Mode images. ECG (B) and inner diameter (C) data are digitized, then R peaks in the ECG time series are identified and used to delimit individual cardiac cycles (red vertical lines in the image, red open dots in the time series). Individual cardiac cycles showing poor tracking or breathing artifacts can be deleted by selecting the “Delete Cycles” button and then clicking anywhere within the area of the cardiac cycle to be discarded. The remaining data can be ensemble averaged to obtain the mean inner diameter waveform over the mean cardiac cycle (D). In the case of poor quality data, the user can specify a few selected cardiac cycles to be considered by selecting the “Select & Average Cycles” button.

Ultrasound images and cine loops capturing central artery motions and invasive pressure data were processed and analyzed using custom or commercial software, the former for semi-automatized detection of luminal diameter, axial length, blood velocity, and blood pressure. In particular, M-Mode sequences and PW Doppler were analyzed to extract luminal diameter and blood velocity waveforms, respectively (Fig 2 and S1 Fig). PW Doppler data for the ATA and IAA were angle corrected to facilitate velocity sampling as parallel as possible to the axis of the vessel (angle of correction at or below 59°). Motivated by prior work [19], we also used automatic traces of Doppler data using the Visualsonics software with options “auto”, “positive”, or “negative” depending on the direction of the flow and quality of the data. M-Mode and PW Doppler sequences were extracted from the Visualsonics software as DICOM stacks that served as inputs to our custom code. Traditionally these measurements are performed manually over a small number of cardiac cycles and limited to systolic and diastolic values. Our graphical user interface (GUI) in Matlab R2016a allows semi-automatic quantification of the data over many cardiac cycles, then extraction of average values of velocity and diameter over a cardiac cycle.

Basic features of our image analysis software include, stitching image sequences to form a larger image containing a continuous series of ultrasound data; automatic detection of the ECG, blood velocities, and inner arterial diameters; exclusion of cycles of data containing artifacts from breathing or other external perturbations; and ensemble averaging to obtain a waveform representing the mean cardiac cycle. Inner arterial diameters were extracted from M-Mode images by detecting luminal edges using a Canny method [20]. The user can adjust the sensitivity threshold and variance of the Gaussian filter to improve edge detection. Blood velocities were extracted by isolating automatic traces based on their RGB values. After extraction, both signals were registered with the ECG and individual cardiac cycles were identified based on locations of R-peaks (red vertical lines overlaid on ultrasound images and red dots overlaid on detected waveforms in Fig 2). Total aortic pulse transit time (PTT)—the time needed for a pulse wave to travel from the aortic root to the iliac bifurcation—was calculated from PW Doppler velocities (S1 Fig). We defined the foot of the blood velocity waveform as the intersection between two tangents [21]: the horizontal tangent intersecting its diastolic minimum and the tangent to the maximum systolic gradient, which was evaluated using a 4th-order accurate central differences scheme (given the constant sampling rate). PTT was calculated as the time lag between the average feet of the ATA and IAA blood velocities, while correcting for minor changes in heart rate.

**Fig 3:**
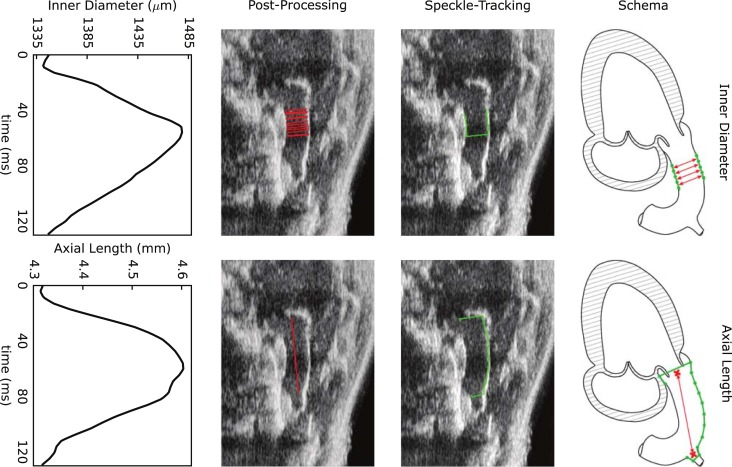
Measurement of biaxial motions of the ATA via B-Mode (first row) and speckle-tracking (second row), with representative post-processing (third row) and resulting mean time-courses (fourth row). Assuming axisymmetric deformations–consistent with the use of *in vitro* biaxial data to specify material properties–cyclic changes in inner diameter and axial length were extracted from long-axis B-Mode cine loops. We used Visualsonics VevoStrain software for speckle-tracking by manually seeding material points on the ATA lumen (green lines). Triplicate analyses on each video allowed us to select the most reliable tracking. Custom post-processing (red lines) allowed visualization of the time-course of lumen and length.

B-Mode cine loops were analyzed using the speckle-tracking algorithm VevoStrain from Visualsonics [22]. Similar to prior work [23], positions of the tracked points were exported to and analyzed within a custom script. Inner diameter and axial length of the ATA were measured from the same long axis cine loop sequence using three-to-six consecutive cardiac cycles depending on image quality. For inner diameter measurements, points were seeded manually on both sides of the visible lumen within one frame while ensuring that they were positioned centrally within the ATA. Such points were subsequently tracked automatically throughout the cine loop (Fig 3, left column) and, assuming axisymmetric deformations, the associated luminal edges were defined by two straight lines; the inner diameter for a given frame was approximated as the mean distance between these two lines. For axial length measurements, points were seeded manually to include the aortic root, the base of the brachiocephalic trunk, and the outer curvature of the ATA (Fig 3, right column). The axial length within a given frame was defined as the linear distance between centroids of the aortic root and base of the brachiocephalic trunk. Appropriate speckle-tracking was evaluated visually and, if deemed unsatisfactory, repeated. Average waveforms of inner diameter and axial length were assumed to capture the overall biaxial motion of the ATA (Fig 3, bottom). In vivo cyclic circumferential and axial stretches, $\lambda_{\vartheta }^{*}$ and $\lambda_{z}^{*}$, were calculated by dividing the systolic inner diameter and axial length by their respective diastolic values.

Intraluminal blood pressure measured in the ATA was analyzed using a custom script that allows the user to select a time interval during which pressure oscillations are stable and devoid of excessive noise or eventual outliers due to breathing or sudden movements of the animal. The script uses R-peaks of the ECG signal to delimit each cardiac cycle, within which distinctive features of the (intraluminal) pressure signal *P*_*i*_(*t*) are extracted. Systolic, diastolic, mean, and pulse pressures were calculated as usual,

$$P_{i}^{sys}=maxP_{i}(t),\;P_{i}^{dias}=minP_{i}(t),\;P_{i}^{mean}=P_{i}^{dias}+\frac{1}{3}P_{i}^{pulse}.$$ (1-3)

Additional outputs include heart rate (inverse of the cardiac period) and body temperature. As appropriate, parameters were averaged across cardiac cycles.

### In vivo arterial biomechanics

The mechanical behavior of an artery is governed by its geometry (luminal radius, wall thickness, and length), intrinsic material properties (which depend on the microstructure and tone generated by contractile smooth muscle), and physiologic loads (mainly intraluminal pressure and axial force, but also the perivascular support from surrounding tissues that manifests largely as a radially directed stress acting inwards on the adventitia). Based on the assumption of negligible basal tone in elastic arteries under anesthetized conditions, we estimated the in vivo material properties of central arteries by combining information on intrinsic passive properties, from in vitro findings [16], and physiologic deformations, from in vivo measurements.

Briefly, consistent with our established in vitro protocols [16,18], mice were euthanized with an intraperitoneal injection of Beuthanasia-D and both a common carotid artery (CAA) and the aorta, from the root to the iliac bifurcation, were excised intact. Cylindrical segments were isolated (ATA, SAA, IAA, CCA), cannulated on micro-pipets, and placed within a custom biaxial testing device at 37^°^C in a Hank’s buffered physiologic solution. Following standard preconditioning (slow cyclic pressurization from 10–140 mmHg at the estimated value of in vivo axial stretch), a series of three cyclic pressure-diameter protocols at different fixed axial stretches (from -5% to +5% of the preferred in vivo value) and four slow, cyclic axial force-length protocols at different fixed pressures (10–140 mmHg) were used to collect passive biaxial mechanical data. The nonlinear and possibly anisotropic behavior of each aortic segment was described separately for each specimen by a pseudoelastic stored energy function *W* [24]

$$W\left(C,M^{i}\right)=\frac{c}{2}\left(I_{C}-3\right)+\sum_{i=1}^{4}\frac{c_{1}^{i}}{4c_{2}^{i}}\left\{\exp\left[c_{2}^{i}{\left(IV_{C}^{i}-1\right)}^{2}\right]-1\right\},$$ (4)

where *I*_*C*_ = *tr***C** and $IV_{C}^{i}=C:{Μ}^{i}⊗{Μ}^{i}$, with $C=diag\left[\lambda_{r}^{2},\lambda_{\vartheta }^{2},\lambda_{z}^{2}\right]$ depending on the principal stretches and **M**^*i*^ representing orientations of locally parallel families of smooth muscle (circumferential) and/or collagen fibers (*i* = 1 (axial), 2 (circumferential), and 3,4 (symmetric diagonal, at angle *α*_*o*_) in a reference configuration). Model parameters $\left[c,c_{1}^{i},c_{2}^{i},\alpha_{o}\right]$ were determined via nonlinear regression of the biaxial data, with best-fit values given by sex and region in S1 Table.

Importantly, with material parameters known from in vitro experiments, in vivo values of mean wall stress and material stiffness can be computed easily given the deformations. In vivo values of the in-plane extensional stretches were computed as *λ*_*ϑ*_ = (*d* + *h*)/(*D* + *H*) and *λ*_*z*_ = *l*/*L*, where *d* and *l* are cyclic mean diameters and lengths extracted from ultrasound, with *D*, *H*, and *L* unloaded dimensions measured in excised samples. In vivo wall thickness *h* was estimated assuming incompressibility [18], thus yielding radial stretches *λ*_*r*_ = *h*/*H* = 1/(*λ*_*ϑ*_*λ*_*z*_). Assuming a near state of plane stress and a deformation gradient tensor **F** = diag(*λ*_*r*_,*λ*_*ϑ*_,*λ*_*z*_), the passive Cauchy stresses can be calculated as [25]

$$\sigma_{ij}=-p\delta_{ij}+2F_{iA}F_{jB}\frac{\partial W}{\partial C_{AB}},$$ (5)

where *p* is a Lagrange multiplier that enforces incompressibility and can be computed from traction boundary conditions and **C** = **F**^*T*^**F** is the right Cauchy-Green tensor. Material stiffness was calculated via an appropriate linearization of the nonlinear mechanical behavior over physiologic deformations [24], namely

$$C_{ijkl}=2\delta_{ik}F_{lA}^{}F_{jB}^{}\frac{\partial W}{\partial C_{AB}}+2\delta_{jk}F_{iA}^{}F_{lB}^{}\frac{\partial W}{\partial C_{AB}}+4F_{iA}^{}F_{jB}^{}F_{kP}^{}F_{lQ}^{}{\frac{\partial^{2}W}{\partial C_{AB}\partial C_{PQ}}|}_{C^{}},$$ (6)

noting that we focused on in-plane values of material stiffness ($C_{\vartheta \vartheta \vartheta \vartheta }$ and $C_{zzzz}$), consistent with our focus on in-plane components of stress (*σ*_*ϑϑ*_ and *σ*_*zz*_). By enforcing quasi-static equilibrium throughout a cardiac cycle [26], we then calculated the external loads acting on each arterial segment (not all of which can be measured), where

$$P_{t}=\sigma_{\vartheta \vartheta }\frac{h}{a},\;f=\sigma_{zz}\pi h(2a+h),$$ (7)

with *P*_*t*_ the transmural pressure (i.e., intraluminal minus perivascular), *f* the total axial force, and *a* the luminal radius. The work done on each arterial segment by such external loads, from systole to diastole, can be computed as

$$U=\int P_{t}\;dV+\int f\;dl,$$ (8)

which was evaluated numerically using a trapezoid rule over the duration of the systolic upstroke. Finally, mean external (perivascular) radial support (i.e., the radial component of the traction vector acting on the external surface, with units of pressure) was quantified for the ATA as a percent of the intraluminal pressure *κ*(%) = (1−*P*_*t*_/*P*_*i*_)×100 at systole and diastole, with *P*_*t*_ and *P*_*i*_ denoting the calculated transmural and measured intraluminal pressures, respectively [26].

### Axial retraction

We sought to validate values of axial stretch identified in vitro based on the energetically-favorable observation of nearly constant axial forces during pressurization [16,27]. Preliminary tests suggested that the in vivo axial stretch could only be determined reliably, via measurements of axial retraction upon excision, in the IAA. Retractions measured in other arterial segments were either too sensitive to surgical manipulations (e.g., movement of the heart for the ATA, position of the neck for the CCA), or the arteries were inclined significantly with respect to the focal plane of the dissection microscope (SAA), thus allowing measurement of projected lengths only. In contrast, the IAA remained in focus over its entire length when imaged via the microscope, and, importantly, had enough axial tethering from side branches to maintain its axial length despite surgical manipulations. After euthanasia, we performed an abdominal incision, removed the viscera, and cleaned the IAA from surrounding tissues (including the adjacent inferior vena cava). The artery was thus isolated, but axially tethered by major branches (renals and iliacs). India ink dots were applied to the adventitial surface, proximally at the level of the left renal artery and distally at the level of the iliac bifurcation, and left to dry for several minutes. The aorta was then excised and placed in physiological saline. Video images of the IAA, in situ (before excision) and ex vivo (after excision), were taken using a CCD camera connected to a stereo miscroscope (S2 Fig). Semi-automatic image analysis using a custom Matlab script quantified the axial retraction. Briefly, the two images were cropped, converted to grayscale, and thresholded to isolate the India ink dots. Length before and after excision was calculated as the distance (in pixels) along the vessel between the centroids of the India ink dots. Axial stretch was computed simply as the axial length in situ divided by the axial length in vitro.

### Statistics

Experimental data are presented as mean±SEM. Two-sided t-tests were used to assess differences between methods used to measure key metrics of interest. One-way analysis of variance (ANOVA) and Tukey’s HSD test for multiple comparisons were used to assess regional differences between the means of mechanical metrics in the ATA, SAA, IAA, and CCA. Differences with *p* < 0.05 were considerate significant.

## Results

**Fig 4:**
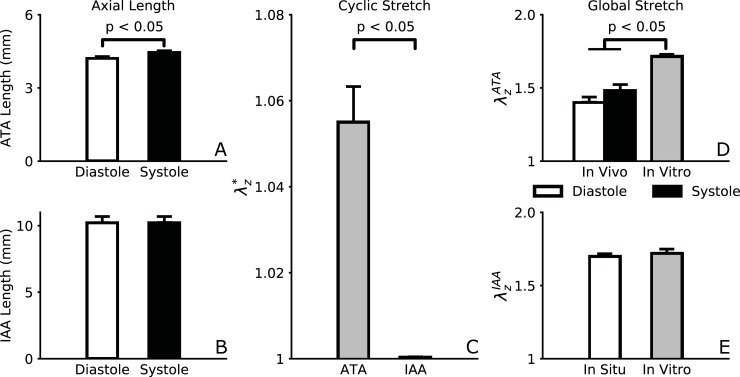
Comparison of axial lengths and deformations of the ATA and IAA measured using B-Mode imaging and speckle-tracking. Values at systole and diastole (A-B) show that the ATA lengthens significantly during systole while the IAA preserves its length, thus leading to a significant difference in the cyclic axial stretches between these two segments (C). Comparison of global (relative to unloaded) axial stretches *in vivo* with the energetically optimal axial stretches measured *in vitro* via biaxial testing (D-E) show that, under anesthesia, the ATA stretches significantly less than it could, while the IAA remains at its energetic optimum.

Noninvasively measured hemodynamics showed that peak velocity decreases as blood flows along the aorta in the healthy adult mouse, with systolic values of 107±7 cm/s in the ATA and 33±4 cm/s in the IAA. The average PTT measured from PW Doppler images between the aortic root and iliac bifurcation was 11±1 ms. Fig 4A and 4B shows cyclic variations in axial length from B-Mode speckle-tracking of the most proximal (ATA) and distal (IAA) segments of the aorta. Measured under anesthesia, and thus depressed cardiac function, ATA length was still significantly greater at systole (4.46±0.07 mm) than diastole (4.22±0.06 mm), yielding a maximum cyclic axial stretch of $\lambda_{z}^{*}$~1.06 (Fig 4C). In contrast, there were no detectable changes in the IAA, suggesting that the ascending aorta undergoes significant axial motions due mainly to its direct connection to the beating heart. Overall axial stretch (relative to the excised unloaded length) measured in vivo was *λ*_*z*_ = 1.46 at systole in the ATA, which was significantly lower than the energetically optimal value (*λ*_*z*_ = 1.72) that was inferred in vitro [16] via the cross-over point in axial force-length tests (Fig 4D). We hypothesize that this difference would become progressively less under ambulatory, exercise, and then “fight or flight” conditions as the heart beats more forcefully and pulls more on the ascending aorta. At the same time, Fig 4E shows that the in situ stretch measured in the IAA just prior to excision matched exactly the optimal value measured in vitro. Noting that cyclic axial stretch can increase elastic energy storage in vivo, these results suggest that axial deformations during the cardiac cycle are a distinct feature of the ascending aorta, which appears to have an “exertion reserve”, whereas other segments (e.g., abdominal aorta) likely operate at a largely unchanging optimum.

**Fig 5:**
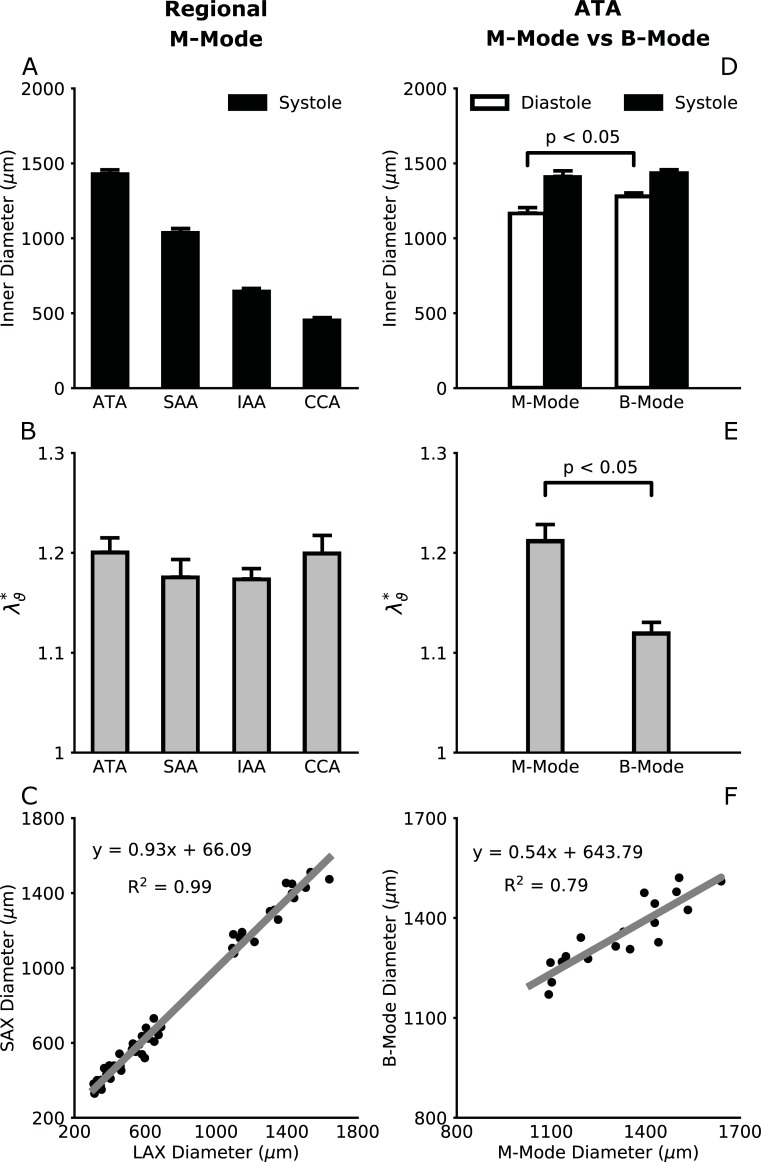
Inner diameters (A,D) and associated cyclic circumferential stretches (B,E) reveal the extent of arterial deformation at systole with respect to diastole. Inner diameters were quantified using our semi-automated methods from regional M-Mode images (left column) and B-Mode cine loops for the ATA (right column). Scatter plots show that long axis (LAX) and short axis (SAX) M-Mode measurements of inner diameter compare well in all regions (C), while LAX M-Mode and B-Mode measurements of inner diameter agree less well in the ATA (F). Note the R^2^ values for the linear fits.

Fig 5A and 5B shows associated values of inner diameter and in vivo cyclic circumferential stretches ($\lambda_{\vartheta }^{*}$) measured regionally via M-Mode imaging and quantified automatically using our GUI (Fig 2). As expected, inner diameter was largest in the ATA, decreasing gradually in the SAA and IAA, and smallest in the CCA. Interestingly, cyclic stretches were nearly identical across these central arteries. Fig 5C compares inner diameters in the ATA, SAA, IAA, and CCA inferred from LAX (abscissa) and SAX (ordinate) images. Linear regression confirmed excellent agreement (i.e., near the line of identity) between automated quantifications of inner diameter from LAX and SAX planes, thus providing further validation of our quantifications. Fig 5D–5F focuses on the ATA and compares values of inner diameter measured using M-Mode and B-Mode imaging. Systolic diameters were similar but diastolic diameters were significantly higher when measured using B-Mode cine-loops (Fig 5D), which implied lower cyclic circumferential stretches (Fig 5E), that is, values closer to the cyclic axial stretches. Fig 5F similarly compares ATA inner diameters measured from LAX views using M-Mode (abscissa) and B-Mode (ordinate) imaging. Despite the strong correlation, the two approaches led to different values. Note, therefore, that B-Mode speckle-tracking is a Lagrangian approach, in which multiple material points are followed throughout several cardiac cycles and their relative positions are used to compute inner diameter and axial length. In contrast, M-Mode is effectively an Eulerian approach, in which the ultrasound beam is fixed and inner diameter at each time is quantified as the distance between points on opposite luminal edges that appear within the field field-of-view (whereby particular edges that are tracked can change if the vessel elongates, bends, or twists with each cardiac cycle). Such differences between B-Mode and M-Mode measurements would likely lead to different results in the ATA alone, where complex motions occur. It is well known, of course, that M-Mode measurements of cyclic deformations in the heart are better closer towards the apex, which moves less.

**Fig 6:**
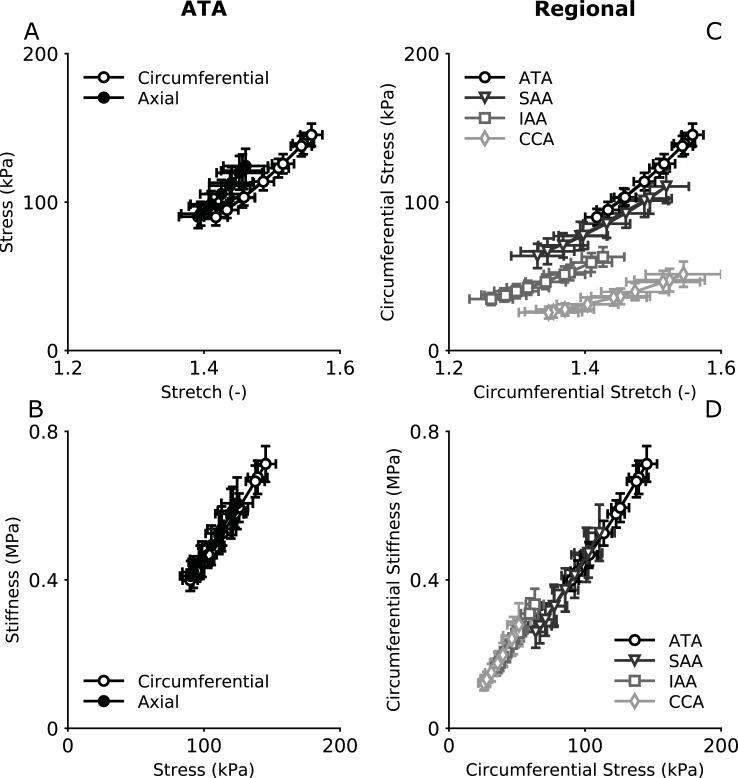
Biaxial material properties visualized as *in vivo* Cauchy stress-stretch (A,C) and stiffness-stress (B,D) relationships for the ATA (A,B) and all regions (C,D). These data suggest that the ATA exhibits a nearly isotropic material behavior *in vivo*, at least under anesthetized conditions Note that circumferential stresses decrease with decreasing arterial caliber while circumferential stiffness appears comparable across all central arteries considered herein, with the linear relation between stiffness and stress expected of an exponential behavior.

Given the in vivo measured biaxial motions (Figs 4 and 5, with axial stretch constant except in the ATA) and the in vitro measured material properties (S1 Table), we computed associated in vivo values of biaxial wall stress (Fig 6, top panels) and material stiffness (Fig 6, bottom panels). Values of Cauchy stress were on the order of 110 kPa and nearly equibiaxial in the ATA (Fig 6A) despite a slightly greater distension than extension. The similarity of biaxial stiffness, on the order of 530 kPa (Fig 6B), further suggested a nearly isotropic in vivo behavior of the ATA. In contrast, the other three regions displayed a highly anisotropic behavior with values of axial stress and stiffness higher than their circumferential counterparts (S3 Fig). A high axial stiffness may provide increased axial mechanical stability against lateral bending. Note, too, that calculated in vivo circumferential stresses tended to vary with luminal caliber, higher in the ATA and lower in the CCA (Fig 6C). The circumferential stiffness-stress relationship yet collapsed along a single line for all four regions despite differences in geometry, material properties, and hemodynamic loading (Fig 6D), thus suggesting an underlying common material behavior of all healthy central arteries, independent of sex (S4 Fig), which justified pooling the in vivo and in vitro data to identify general trends in the adult wild type mouse. Finally, that the biaxial stiffness-stress relationship was nearly linear in all regions reflects an underlying exponential-type behavior (Eq 4; cf. [28]).

**Fig 7:**
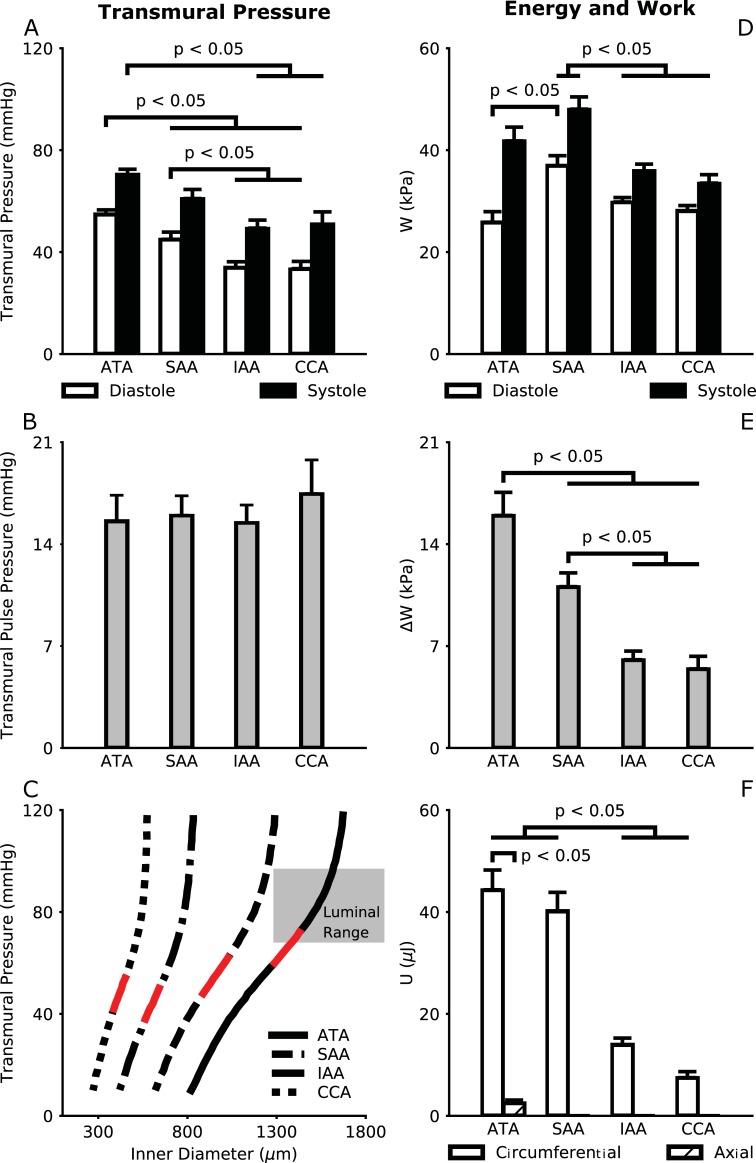
Measurements of in vivo motions and in vitro properties allow one to estimate regionally varying transmural pressures at systole and diastole (A) and associated pulse pressures (B). The effect of perivascular support can be visualized for the ATA (solid line), SAA (dashed line), IAA (dash-dot line), and CCA (dotted line), with superimposed *in vivo* ranges shown as red segments (C). Luminal blood pressure was only measured directly in the ATA, shown by the shaded region. Elastic energy stored at *in vivo* values of systolic and diastolic pressures can also be calculated relative to an unloaded configuration (D). Part of this energy is stored *in vivo* over a cardiac cycle, which can be used to work on the blood during diastole (E). While absolute energy storage is highest in the SAA, cyclic energy storage is highest in the ATA. Quantification of the work done by external loads (F) suggests that, despite being much smaller than its circumferential counterpart, axial work contributes to energy storage by the ATA even under anesthetized conditions and thereby influences its function *in vivo*.

Fig 7A shows calculated regional variations in transmural pressure (based on in vivo deformations and in vitro material properties), with systolic and diastolic values highest in the ATA, less in the SAA, and lower yet in the IAA and CCA where the pressures assume similar values. Interestingly, the calculated transmural pulse pressures were similar across all central arteries (Fig 7B), which mirrors the M-mode measured constant cyclic stretches (Fig 5B). Fig 7C shows average pressure-diameter behaviors measured in vitro [16] in the four regions of interest with a superimposed range of luminal diameters (red segments) measured in vivo. Importantly, this representation suggests that central arteries may operate in vivo near the inflection point of the pressure-diameter curve, which results in the highest possible compliance, at least under anesthesia. Fig 7D shows regional variations in elastic stored energy *W*, with absolute energy storage (i.e., with respect to an unloaded configuration) highest in the SAA. When accounting for actual biaxial motions (if present) and inferred effects of perivascular support in all regions, we found that all segments store less energy in vivo than previously estimated when imposing a constant axial length and blood pressures measured in conscious animals via tail-cuff (cf. Fig 4A in [16]). While systolic energy storage (*W*^*sys*^) was highest in the SAA, Fig 7E shows that cyclic energy storage (Δ*W* = *W*^*sys*^−*W*^*dia*^) was highest in the ATA. This finding correlates well with the percent elastin within the wall (highest in the ATA) and the biaxial loading of the ATA. The circumferential work performed by blood pressure on the wall was similar for the ATA and SAA, but lower in the IAA and CCA (Fig 7F). Although the axial work was significantly lower than the corresponding circumferential work (under anesthetized conditions), it increased the cyclic energy storage of the ATA beyond that of the SAA. These data support the notion that axial motions of the ATA contribute to its physiologic function, which in turn influences cardiac function via direct and indirect ventricular-vascular coupling. The axial contribution would be expected to be even higher in cases of exercise and exertion.

**Fig 8:**
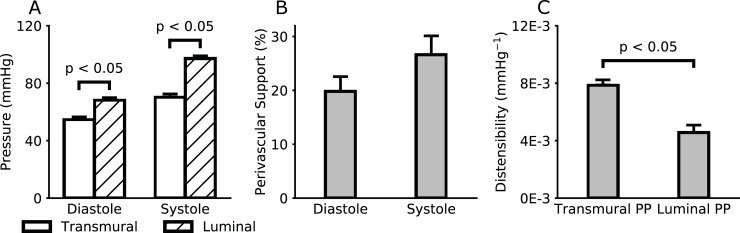
Luminal pressures measured directly via a Millar catheter are higher than transmural pressures estimated using *in vivo* measured motions and *in vitro* measured material properties (A). Such differences suggest that a portion of the pressure-loading is transferred to perivascular tissues (B), thus decreasing the net outward pressure acting on the arterial wall, which affects associated calculations of distensibility *D* (C), with that based on transmural pressure more representative of the in vivo situation.

Finally, recall from Fig 7C the partial overlap between the range of intraluminal pressures measured directly in the ATA (grey box) and that inferred from wall motions (vertical projection of the red line). This difference in pressures is assumed to be due largely to the mechanical action of surrounding tissues, which is typically neglected. Millar catheter measurements revealed in vivo values of blood pressure in the ATA of $P_{i}^{dia}=68\pm 2mmHg$, $P_{i}^{sys}=97\pm 2mmHg$, $P_{i}^{mean}=78\pm 2mmHg$, and $P_{i}^{pulse}=29\pm 1mmHg$, with an average heart rate of 443±19 bpm (under anesthesia). Fig 8A compares these intraluminal values with transmural values (i.e., intraluminal minus perivascular) estimated from in vivo measured deformations and in vitro measured material properties, which yielded $P_{t}^{dia}=55\pm 2mmHg$, $P_{t}^{sys}=70\pm 2mmHg$, $P_{t}^{mean}=60\pm 2mmHg$, and $P_{t}^{pulse}=16\pm 2mmHg$. Differences between intraluminal and transmural pressures (with *P*_*t*_ = *P*_*i*_ − *P*_*p*_) suggested mean levels of perivascular support (*κ*) from 20% (at diastole) to 27% (at systole) of the intraluminal pressure in the ATA (Fig 8B). Although *κ* did not change much throughout the cardiac cycle in these wild-type mice under anesthesia, it could increase with increases in intraluminal pressure due to the nonlinear mechanical behavior of most soft tissues. That is, perivascular support could be greater if measured under conditions of exertion or hypertension. Of course, perivascular support plays an implicit but key role in the aforementioned clinical metric of arterial compliance known as distensibility *D*. Because intraluminal pulse pressure is always higher than its transmural counterpart, this leads to lower values of distensibility for equal changes in luminal diameter (Fig 8C). Caution is warranted, therefore, when comparing values of distensibility measured in vivo versus those measured on isolated samples in vitro (without perivascular support).

## Discussion

We previously emphasized the importance of consistent in vitro experimental methods and data analysis [18] and showed that consistent biomechanical phenotyping can identify similarities and delineate differences across mouse models [16,29–31]. The proposed in vivo protocols and methods of semi-automated analysis complement our prior in vitro methods, noting that true insight into central artery biomechanics and ventricular-vascular coupling requires a melding of in vitro and in vivo data. Specifically, to improve reproducibility and increase throughput, we developed semi-automated approaches to analyze M-Mode and PW Doppler images with the goal of extracting luminal diameter and blood velocity waveforms over many cardiac cycles (Fig 2 and S1 Fig). M-Mode tracking allows direct comparisons of luminal diameters from central arteries of different caliber (by maintaining the same spatial and temporal resolutions) while the temporal resolution of B-Mode cine loops decreases as the field-of-view increases. The latter trade-off still allows optimal B-Mode tracking in short segments such as the ATA. Indeed, we suggest that the Lagrangian foundation of B-Mode tracking is preferred for the ATA, which experiences complex motions over a cardiac cycle. Rather than develop new tracking methods for B-Mode imaging, we used available VevoStrain software to compute biaxial deformations of the ATA from surface features in LAX views under the assumption of axisymmetric motions (Fig 1).

Using these various methods, three key findings emerged on central artery biomechanics. First, the normal healthy ATA is biomechanically distinct among central arteries for it experiences marked circumferential distensions and axial extensions with each beat of the heart (Figs 4 and 5) while other regions experience limited macroscopic axial motions. Consistent with the biaxial strains experienced by the ATA, it alone exhibited a near isotropic mechanical behavior in vivo (Fig 6) and it stored the most elastic energy during a cardiac cycle (Fig 7), which could be used during diastole both to augment blood flow (including retrograde flow needed to perfuse the heart during diastole) and to aid diastolic filling of the LV (by helping to lift the base of the heart). Cyclic axial extensions contributed to this energy storage under anesthetized conditions though not at the theoretically optimal value (cf. Fig 4). By comparing in vitro and in vivo values, we hypothesize the existence of an “exertion reserve” whereby axial stretch can progressively increase in the ATA from anesthetized to normal resting, exercise, and ultimately fight or flight conditions, thus enabling increasingly greater stored energy to be available to augment blood flow or diastolic filling in cases of greater demand. Notwithstanding these important distinctions of the ATA, we also found qualitatively, and in many cases quantitatively, similar behaviors across the four regions examined. Circumferential cyclic strain (Fig 5), circumferential material stiffness (Fig 6), and transmural pulse pressure (Fig 7) were comparable across regions, independent of sex, suggesting the existence of some general homeostatic targets. Although associated calculations were based on the assumption of no cyclic axial stretching in the SAA, IAA, and CCA (segments that are assumed to mature to optimal stretches during somatic growth since elastic fibers have a long half-life; [25]), axial motions have nevertheless been reported in the CCA in humans and mice [32]. These motions are usually multiphasic and their physiological significance unclear [33,34], hence there is need for further investigation.

Second, including effects of perivascular support, we found that the Cauchy stress-stretch behavior is only mildly nonlinear over a cardiac cyclic (Fig 6C) and there appears to be a common material stiffness-stress relation across all regions (Fig 6D). The former supports the use of a “Small on Large” theory [24] to compute material stiffness for use in fluid-solid-interaction simulations of the hemodynamics. Related to the latter, it was surprising that the in vivo value of stress tended to decrease with decreasing caliber, which tends to associate with less elastin and more smooth muscle [16,35]. It would be useful to quantify possible regional differences in basal tone and how such differences could affect local stresses, noting that basal tone, if present, would be expected to be low in exercise and exertion to reduce the workload of the heart, consistent with increased flow-induced wall shear stress promoting endothelial cell nitric oxide production.

Third, calculated values of the mean perivascular radial traction on the ATA were on the order of 23% of the intraluminal blood pressure, that is, ~3 kPa (Fig 8). Although this inference was possible only in the ATA, where the Millar catheter measured intraluminal pressure directly, calculated values of transmural pulse pressure (under anesthetized conditions) were similar from region-to-region (Fig 7B), not accounting for differences in the pulse wave along the aorta which could be affected by possible regional differences in smooth muscle cell tone. There is, therefore, a pressing need for additional experimental methods to delineate effects of smooth muscle tone locally versus globally (e.g., total peripheral resistance and thus mean blood pressure) and to employ complementary fluid-solid-interaction simulations to understand better any regional differences in mechanical loading [25]. In vivo measurements of deformations will remain fundamental to any estimation of perivascular tethering, and future studies should consider even more complex motions of the ATA, including bending and twisting. Nonetheless, it appears that all central arteries in healthy wild-type mice operate (again, under anesthetized conditions) within a highly compliant portion of the pressure-diameter curve (Fig 7C), hence allowing considerable distension upon increased pressurization (at least from anesthetized values). This finding affects all in vivo calculations of mural stress and material stiffness (Fig 6) as well as energy storage (Fig 7D–7F).

Many of the present results provide important baseline values for adult wild-type controls (on a C57BL/6 x 129/SvEv background) to which future results for different mouse models can and should be compared. Differences between wild-type male and female animals were minimal and limited to geometrical metrics (not shown), while material metrics were indistinguishable between the two sexes (S4 Fig). Moreover, anesthetized heart rates measured herein were comparable with many prior reports, ranging from 400 to 500 bpm, though lower than in some reports. Importantly, isoflurane decreases total peripheral resistance [36], which suggests a vasodilatory function in addition to other effects. This observation coupled with the recent finding that systemic administration of vasoactive substances in anesthetized (urethane) mice does not alter aortic stiffness [37] supports our assumption of a primarily passive central arterial state during in vivo measurements, though greater understanding is needed. Because slow cyclic in vitro testing reveals only slight hysteresis in Cauchy stress-stretch results [16], our constitutive model focused on the pseudoelastic response during unloading to estimate the elastic energy available to do work. Future in vitro studies should consider viscoelastic constitutive models based on cyclic testing over normal laboratory as well as in vivo relevant ranges of pressure, the latter at in vivo rates of loading. Similarly, there is a pressing need for complementary hemodynamic simulations given the difficulty of measuring cyclic pressures locally throughout the murine vasculature [38], which can vary from region to region [39]. Finally, we did not include histological data that show regional differences in composition since such results are available elsewhere [16,35,40]. Although it is not possible to know precisely whether adventitial collagen has been inadvertently removed when eliminating loose perivascular tissue, results obtained by different investigators in our laboratory have yielded similar values of wall thickness and medial:adventitial ratios. Histological studies of the aorta from whole perfused mice would be needed to verify normal medial:adventitial ratios.

In conclusion, the widespread availability of genetically altered, pharmacologically treated, and surgically modified mouse models necessitates consistent methods of experimentation and analysis to facilitate comparisons across models and laboratories, thereby increasing the overall information available. Similarly, the necessity of melding in vitro and in vivo data to understand fully the associated mechanobiology and pathophysiology necessitates consistent and complementary methods. We presented new protocols and methods of analysis for melding diverse data for central arteries in the mouse, which yielded important control data for future comparisons as well as new insight into the extent of perivascular tethering and the unique function of the ascending aorta despite many characteristics that are shared in common with other central arteries. There is now a need to study in vivo aortic mechanics similarly in the many mouse models of disease and its treatment.

## Supporting information

S1 Fig Graphical user interface (GUI) for semi-automated pulse wave (PW) Doppler data analysis. Note that the Canny filter parameters are inactive since detection of blood velocity waveforms is based on identified envelopes of the raw PW Doppler data exported from the Visualsonics software. Compare with Fig 2 in the main text.
(PDF)

S2 Fig Example of axial retraction upon excision of a wild type infrarenal abdominal aorta (IAA), which can be imaged in situ and ex vivo in its entirety using a dissection microscope at the same magnification and has enough axial tethering in situ from side branches to maintain its axial length despite surgical manipulation. (PDF)

S3 Fig In vivo biaxial cauchy stress-stretch (top row) and stiffness-stress (bottom row) relations are shown for the four different arterial regions: ascending thoracic aorta (ATA), suprarenal abdominal aorta (SAA), infrarenal abdominal aorta (IAA), and common carotid artery (CCA). Note the assumed constancy of axial stretch in the SAA, IAA, and CCA during a cardiac cycle (top row), which is inferred in vitro as the cross-over point in axial force-length tests at different constant transmural pressures. Note, too, the decreasing values of stress with decreasing arterial caliber (top row, left to right), with apparent isotropic behaviors only in the ATA (left column), which alone experiences significant biaxial motions during a cardiac cycle.
(PDF)

S4 Fig In vivo circumferential cauchy stress-stretch (top row) and stiffness-stress (bottom row) relations are shown for the four different arterial regions (ATA, SAA, IAA, CCA) for both sexes. Note that all such material metrics (stretch, stress, and stiffness) are indistinguishable between adult male and female wild type animals. It was for this reason that data in the main text were pooled.
(PDF)

S1 TableBest-fit values of the constitutive parameters in the stored energy function were obtained via nonlinear regression analysis of mean biaxial data from seven different protocols for eight individual groups defined by sex and vascular region (*n* = 5 per group).Parameters for male (M) and female (F) mice were used in combination with ultrasound measurements of axial length and inner diameter from animals of matching sex. Later, in vivo biomechanical metrics were pooled due to lack of significant sex-dependent differences (cf. S Fig 4). Note how the model parameters display similar regional trends in both male and female animals; the RMSE (root mean square of the error) indicates the goodness of theoretical fit to data. Generally, the parameter *c*, which is meant to capture elastin-dominated isotropic behaviors, decreased along the aorta consistent with histological variations in elastin content. Note, too, that the parameters for the diagonal fiber families ($c_{k}^{3,4}$) tend to change systematically along the length of the aorta, with the associated angle (*α*_*o*_) closest to 45 degrees (consistent with more isotropic behavior) in the ATA. Yet, we caution against over interpreting values of individual parameters. Rather, we favor interpreting overall consequences of each set of best-fit parameter values as reflected in the calculation of biaxial wall stress, material stiffness, and elastic energy storage since the constitutive model is structurally-motivated but phenomenological.(PDF)
