# Supplementary material for: Combining in vivo and in vitro biomechanical data reveals key roles of perivascular tethering in central artery function
Source: PLoS One. 2018 Sep 7;13(9):e0201379. doi: 10.1371/journal.pone.0201379 (PMC6128471; doi:10.1371/journal.pone.0201379)
Supplement: S1 Fig — Note that the Canny filter parameters are inactive since detection of blood velocity waveforms is based on identified envelopes of the raw PW Doppler data exported from the Visualsonics software. Compare with Fig 2 in the main text. (PDF) [file pone.0201379.s001.pdf]

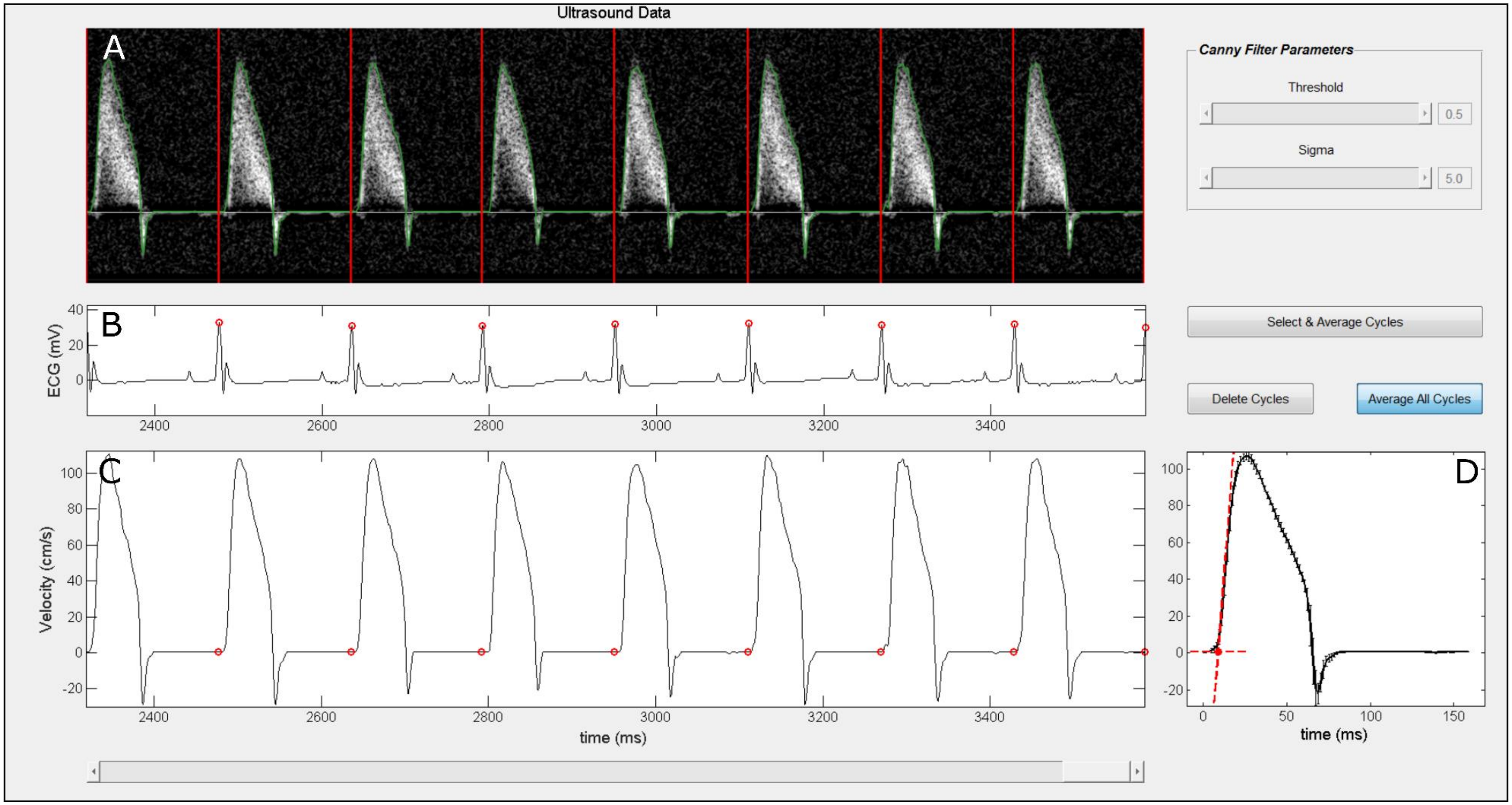

S1 Fig. Graphical user interface (GUI) for semi-automated Pulse Wave (PW) Doppler data analysis. Note that the Canny filter parameters are inactive since detection of blood velocity waveforms is based on identified envelopes of the raw PW Doppler data exported from the Visualsonics software. Compare with Fig 2 in the main text.
