# Supplementary material for: Combining in vivo and in vitro biomechanical data reveals key roles of perivascular tethering in central artery function
Source: PLoS One. 2018 Sep 7;13(9):e0201379. doi: 10.1371/journal.pone.0201379 (PMC6128471; doi:10.1371/journal.pone.0201379)
Supplement: S2 Fig — (PDF) [file pone.0201379.s002.pdf]

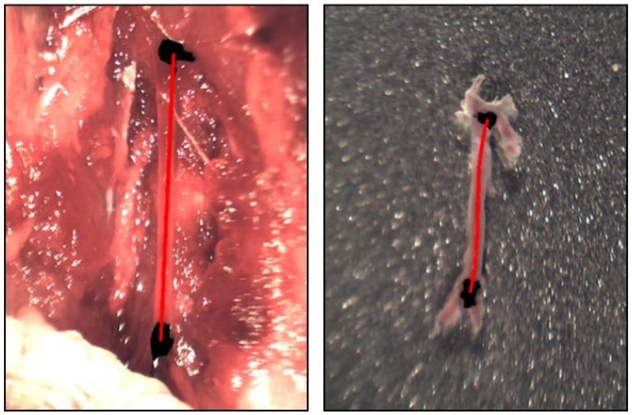

S2 Fig. Example of axial retraction upon excision of a wild type infrarenal abdominal aorta (IAA), which can be imaged in situ and ex vivo in its entirety using a dissection microscope at the same magnification and has enough axial tethering in situ from side branches to maintain its axial length despite surgical manipulation.
