# Supplementary material for: Combining in vivo and in vitro biomechanical data reveals key roles of perivascular tethering in central artery function
Source: PLoS One. 2018 Sep 7;13(9):e0201379. doi: 10.1371/journal.pone.0201379 (PMC6128471; doi:10.1371/journal.pone.0201379)
Supplement: S3 Fig — Note the assumed constancy of axial stretch in the SAA, IAA, and CCA during a cardiac cycle (top row), which is inferred in vitro as the cross-over point in axial force-length tests at different constant transmural pressures. Note, too, the decreasing values of stress with decreasing arterial caliber (top row, left to right), with apparent isotropic behaviors only in the ATA (left column), which alone experiences significant biaxial motions during a cardiac cycle. (PDF) [file pone.0201379.s003.pdf]

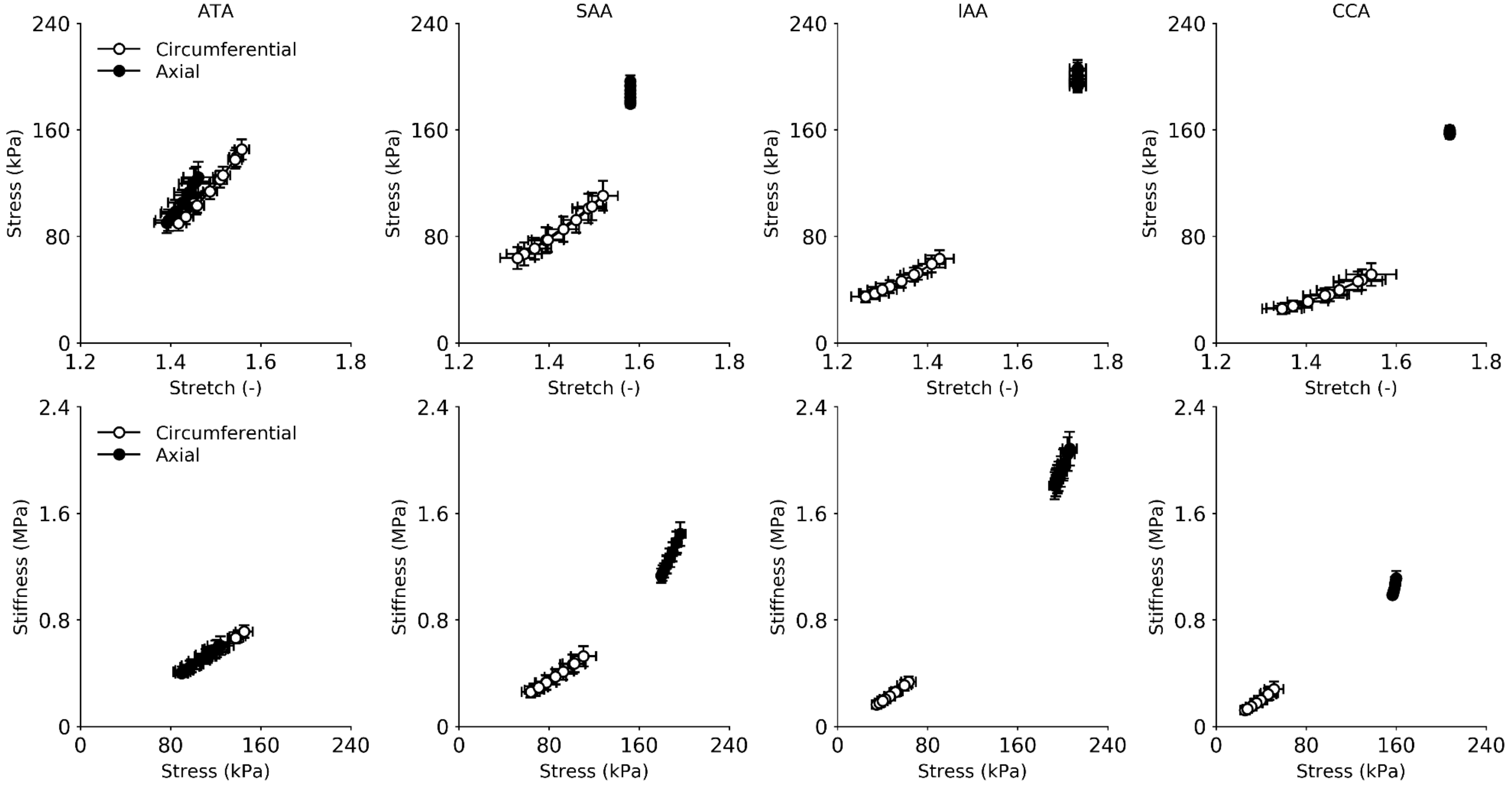

S3 Fig. In vivo biaxial Cauchy stress-stretch (top row) and stiffness-stress (bottom row) relations are shown for the four different arterial regions: ascending thoracic aorta (ATA), suprarenal abdominal aorta (SAA), infrarenal abdominal aorta (IAA), and common carotid artery (CCA). Note the assumed constancy of axial stretch in the SAA, IAA, and CCA during a cardiac cycle (top row), which is inferred in vitro as the cross-over point in axial force-length tests at different constant transmural pressures. Note, too, the decreasing values of stress with decreasing arterial caliber (top row, left to right), with apparent isotropic behaviors only in the ATA (left column), which alone experiences significant biaxial motions during a cardiac cycle.
