# Supplementary material for: Combining in vivo and in vitro biomechanical data reveals key roles of perivascular tethering in central artery function
Source: PLoS One. 2018 Sep 7;13(9):e0201379. doi: 10.1371/journal.pone.0201379 (PMC6128471; doi:10.1371/journal.pone.0201379)
Supplement: S4 Fig — In vivo circumferential cauchy stress-stretch (top row) and stiffness-stress (bottom row) relations are shown for the four different arterial regions (ATA, SAA, IAA, CCA) for both sexes. Note that all such material metrics (stretch, stress, and stiffness) are indistinguishable between adult male and female wild type animals. It was for this reason that data in the main text were pooled. (PDF) [file pone.0201379.s004.pdf]

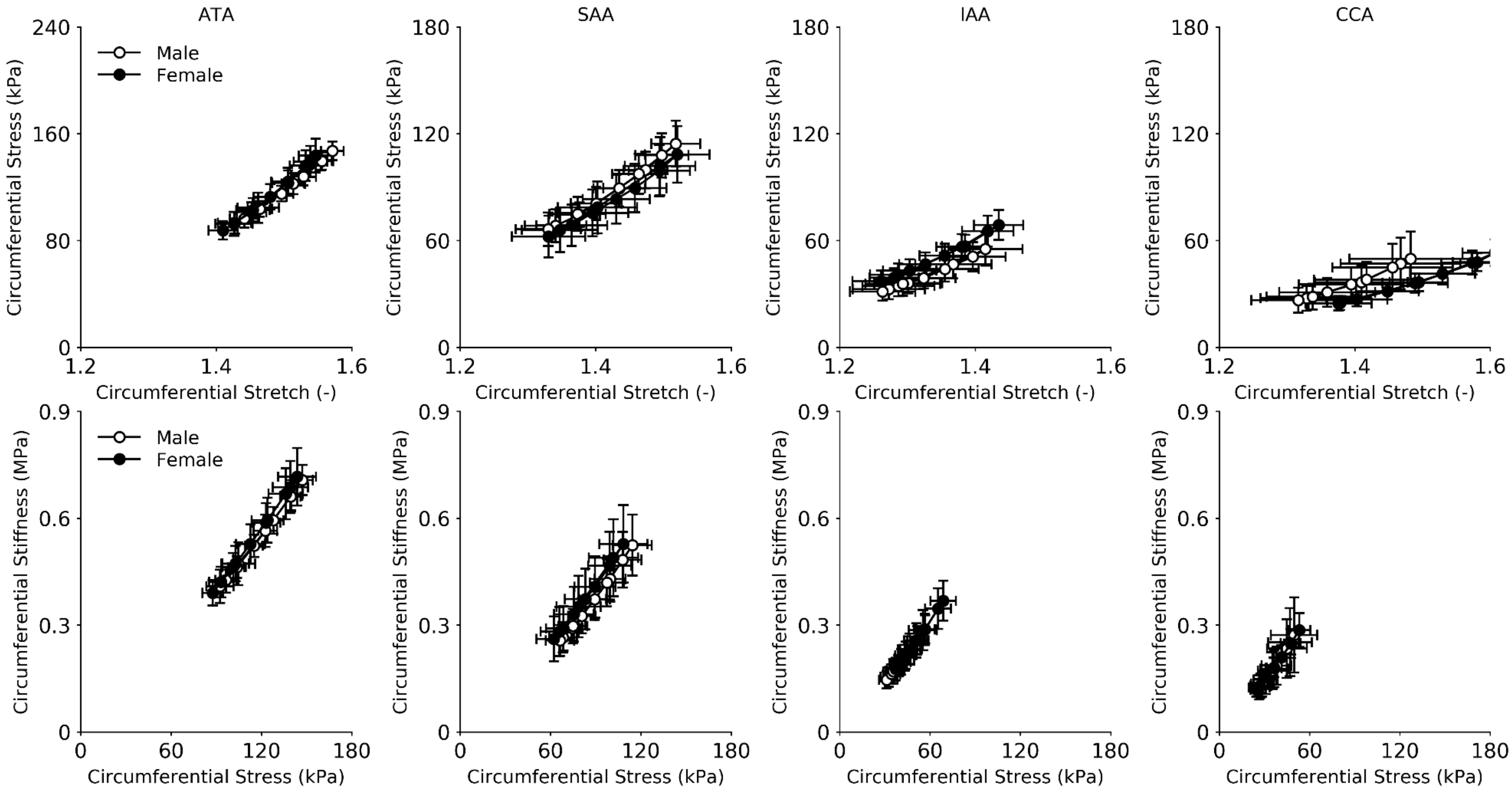

S4 Fig. In vivo circumferential Cauchy stress-stretch (top row) and stiffness-stress (bottom row) relations are shown for the four different arterial regions (ATA, SAA, IAA, CCA) for both sexes. Note that all such material metrics (stretch, stress, and stiffness) are indistinguishable between adult male and female wild type animals. It was for this reason that data in the main text were pooled.
