# Supplementary material for: Combining in vivo and in vitro biomechanical data reveals key roles of perivascular tethering in central artery function
Source: PLoS One. 2018 Sep 7;13(9):e0201379. doi: 10.1371/journal.pone.0201379 (PMC6128471; doi:10.1371/journal.pone.0201379)
Supplement: S1 Table — Parameters for male (M) and female (F) mice were used in combination with ultrasound measurements of axial length and inner diameter from animals of matching sex. Later, in vivo biomechanical metrics were pooled due to lack of significant sex-dependent differences (cf. S Fig 4). Note how the model parameters display similar regional trends in both male and female animals; the RMSE (root mean square of the error) indicates the goodness of theoretical fit to data. Generally, the parameter c, which is meant to capture elastin-dominated isotropic behaviors, decreased along the aorta consistent with histological variations in elastin content. Note, too, that the parameters for the diagonal fiber families (ck3,4) tend to change systematically along the length of the aorta, with the associated angle (αo) closest to 45 degrees (consistent with more isotropic behavior) in the ATA. Yet, we caution against over interpreting values of individual parameters. Rather, we favor interpreting overall consequences of each set of best-fit parameter values as reflected in the calculation of biaxial wall stress, material stiffness, and elastic energy storage since the constitutive model is structurally-motivated but phenomenological. (PDF) [file pone.0201379.s005.pdf]

S1 Table. Best-fit values of the constitutive parameters in the stored energy function were obtained via nonlinear regression analysis of mean biaxial data from seven different protocols for eight individual groups defined by sex and vascular region (n = 5 per group; adapted from Ferruzzi et al., 2015). Parameters for male (M) and female (F) mice were used in combination with ultrasound measurements of axial length and inner diameter from animals of matching sex. Later, in vivo biomechanical metrics were pooled due to lack of significant sex-dependent differences (cf. S3 Fig). Note how the model parameters display similar regional trends in both male and female animals; the RMSE (root mean square of the error) indicates the goodness of theoretical fit to data. Generally, the parameter c, which is meant to capture elastin-dominated isotropic behaviors, decreased along the aorta consistent with histological variations in elastin content (Ferruzzi et al., 2015). Note, too, that the parameters for the diagonal fiber families ($c_k^{3,4}$) tend to change systematically along the length of the aorta, with the associated angle (α_o) closest to 45 degrees (consistent with more isotropic behavior) in the ATA. Yet, we caution against over interpreting values of individual parameters. Rather, we favor interpreting overall consequences of each set of best-fit parameter values as reflected in the calculation of biaxial wall stress, material stiffness, and elastic energy storage since the constitutive model is structurally-motivated but phenomenological.

Sex	*Region*	c *(kPa)*	c_1^1 *(kPa)*	c_2^1	c_1^2 *(kPa)*	c_2^2	$c_1^{3,4}$ *(kPa)*	$c_2^{3,4}$	α_0 *(deg)*	*RMSE*
M	ATA	26.159	16.653	0.080	14.881	0.081	4.729	0.433	44.055	0.079
	SAA	29.056	22.659	0.119	10.194	0.136	0.420	1.350	31.233	0.058
	IAA	12.634	16.888	0.162	10.748	0.124	0.035	1.682	29.394	0.077
	CCA	8.073	17.084	0.069	8.854	0.056	1.97×10^{-5}	2.643	35.089	0.085
F	ATA	23.329	16.799	0.037	15.138	0.102	4.189	0.435	39.887	0.081
	SAA	24.630	22.894	0.060	10.145	0.118	0.313	1.324	30.143	0.073
	IAA	13.388	6.844	0.227	10.946	0.144	0.264	0.856	34.263	0.114
	CCA	6.056	18.348	0.084	7.402	0.052	9.49×10^{-4}	1.981	33.365	0.098
